# Water quality in reference watersheds in the United States: A compilation and analysis of small watershed data

**DOI:** 10.1002/jeq2.70176

**Published:** 2026-04-29

**Authors:** R. Daren Harmel, Devendra Amatya, Stephen Sebestyen, Ge Sun, Joshua Mott, Merilynn Schantz, Johnny Boggs, Peter Caldwell, John Campbell, Aaron Hird, Elizabeth T. Keppeler, Ben Rau, Sherri L. Johnson, Doug Smith

**Affiliations:** ^1^ USDA‐ARS, Center for Agricultural Resources Research Fort Collins Colorado USA; ^2^ USDA Forest Service, Center for Forest Watershed Research Cordesville South Carolina USA; ^3^ USDA Forest Service, Northern Research Station Grand Rapids Minnesota USA; ^4^ USDA Forest Service, Eastern Forest Environmental Threat Assessment Center Durham North Carolina USA; ^5^ USDA‐ARS, Soil Management and Sugar Beet Research Unit Fort Collins Colorado USA; ^6^ USDA‐ARS, Grassland, Soil, and Water Research Laboratory Temple Texas USA; ^7^ USDA Forest Service, Coweeta Hydrologic Laboratory, Southern Research Station Otto North Carolina USA; ^8^ USDA Forest Service, Hubbard Brook Experimental Forest, Northern Research Station Durham New Hampshire USA; ^9^ USDA‐NRCS, Resource Inventory and Assessment Division Lincoln Nebraska USA; ^10^ USDA Forest Service, Caspar Creek Experimental Watershed, Pacific Southwest Research Station Fort Bragg California USA; ^11^ USDA Forest Service, Fernow Experimental Forest, Northern Research Station Parsons West Virginia USA; ^12^ USDA Forest Service, Pacific Northwest Research Station Corvallis Oregon USA

## Abstract

The natural background contribution from grasslands and forest lands is important to consider in research and management to address the contribution of agricultural, industrial, and urban lands to water quality degradation. To our knowledge, no study has compiled and analyzed reference water quality from small reference grasslands and forests even though land use export coefficients for background water quality are assigned at that scale in decision support tools and models, total maximum daily load projects, and comparative analysis. Thus, our major objective was to summarize nitrogen (N), phosphorus (P), and sediment loads in runoff from grassland and forested reference watersheds. Measured annual nutrient loads were available from 13 grassland and nine forest reference sites in 12 North American Level II ecoregions. The grassland reference sites were relatively arid with annual runoff <353 mm (average runoff coefficient = 0.11), and forest reference sites were humid with runoff ranging from 108 to 1274 mm (average runoff coefficient = 0.34). Grassland reference watersheds tended to have higher annual sediments loads (>300 kg/ha), while forest reference watersheds tended to have higher dissolved N loads. This research provides valuable summary results and initial comparisons related to reference water quality across the United States that can serve as a benchmark to compare how anthropogenic activities affect this vital ecosystem service.

## Plain Language Summary

The natural background contribution from grasslands and forest lands is important to consider in research and management. To our knowledge, no study has compiled and analyzed this “reference” water quality from small natural grasslands and forests. Thus, our objective was to summarize nitrogen (N), phosphorus (P), and sediment loads in runoff from these reference watersheds. Measured annual nutrient loads were compiled from 13 grassland and nine forest reference sites across 12 North American regions. The grassland reference sites were relatively arid with mean annual runoff below 353 mm, and forest reference sites were humid with runoff ranging from 108 to 1274 mm. Grassland reference watersheds tended to have higher annual sediments loads, while forest reference watersheds tended to have higher N and P loads. This research provides valuable summary results and initial comparisons that can serve as a benchmark to compare how human activities affect this vital ecosystem service.

**Abbreviations**
BMP: best management practice
MANAGE: Measured Annual Nutrient loads from Agricultural Environments
PET: potential evapotranspiration
TMDL: total maximum daily load

## 1 INTRODUCTION

Despite concerted efforts across multiple sectors in the United States, excess nutrient and sediment runoff continues to adversely impact water resources leading to aquatic ecosystem degradation, increased water treatment costs, and high‐profile litigation (e.g., Christianson et al., 2018; Kleinman & Harmel, 2023; McLellan et al., 2015; Scott et al., 2011). The natural contribution of sediment and nutrient runoff from undisturbed or minimally disturbed land, which is often called a reference site or reference watershed, is an important consideration in research and management to address agricultural, industrial, and urban sources and assess ecological conditions (Hawkins et al., 2019). According to USEPA (2001), reference watersheds are pristine or minimally impacted with few, if any, anthropogenic inputs or modifications. Similarly, Stoddard et al. (2006) state that a “reference condition” can refer to a variety of biological conditions, including historical, least disturbed, minimally disturbed, and best attainable, and explain that reference sites should be ecologically organized and held stable with conservation management, whereas reference (benchmark or natural) water quality has been analyzed at the river basin scale and at the small headwater watershed scale for forested lands, sediment and nutrient runoff from grassland reference watersheds has not been compiled and analyzed.

A reference condition allows us to judge if the measured condition of the resource differs from a desired, expected, or previous condition (Hawkins et al., 2019). Argerich et al. (2013) highlighted the critical value of long‐term, uninterrupted stream chemistry monitoring at a network of sites across the United States to elucidate patterns of change in nutrient concentrations at minimally disturbed forested sites. Neary et al. (2012) and Amatya et al. (2016) note that forest reference watersheds provide valuable insight into ecohydrological processes in relatively undisturbed forest ecosystems and that they are well suited for documenting and detailing baseline (benchmark) conditions to address emerging issues. Ryan (2022) further highlighted the value of forest reference watersheds to inform development of water quality criteria and assessment of stream biological health. Das et al. (2024) underscored the value of prairie reference sites that exhibit ecologically organized attributes within a local landscape context for comparative analysis and for establishing a standard to assess ecosystem changes and dynamic soil properties. Reference sites can benefit from conservation management to maintain resilience and can continue evolving, allowing them to serve as viable and dynamic “constants” and as “living benchmarks.”

Reference water quality and related watershed processes have been comprehensively evaluated across large (river basin) scales. Studies such as Meybeck (1982) and R. A. Smith et al. (2003) have analyzed natural background concentrations of carbon (C), nitrogen (N), and phosphorus (P) in “unpolluted” streams and large rivers pointing to the potential of adverse anthropogenic impact at regional‐ to continental‐scales and the importance of understanding background levels. R. A. Smith et al. (2003) concluded that nutrient loads can vary by more than two orders of magnitude at undeveloped large stream and river reference sites. Ryan (2022) reviewed existing datasets from 17 USDA Forest Service‐affiliated experimental forests and ranges and highlighted the value of research networks containing multiple reference sites in addressing large‐scale issues that span ecoregions. These experimental forests are also representative of climate patterns, ecosystem structure, and ecosystem functions at regional scales (Xiao et al., 2024).

Substantial research has also been conducted on small‐scale forest reference sites (Sebestyen et al., 2025). These watersheds (typically <5 km^2^) permit characterization and understanding of ecosystem processes within relatively simple, homogeneous biological and physical settings but are large enough to evaluate more complex processes and element cycling (Argerich et al., 2013; Huntington et al., 1996). Jones et al. (2012) and Amatya et al. (2016, 2025) have analyzed trends in flow, hydrometeorological influences on hydrologic processes, and water quality in small, forested reference watersheds (<10 km^2^). Based on analysis of data from 10 US Forest Service Experimental Forests, Amatya et al. (2025) found that site specific factors (e.g., climate, land use/land cover changes, and watershed characteristics) determine the response of streams to precipitation inputs, which is consistent with Argerich et al. (2013) who reported that trends in N concentrations differed among neighboring catchments within several experimental forests, suggesting the importance of catchment‐specific factors in determining nutrient exports. Analyzing long‐term record from 35 headwater basins in United States and Canada, Jones et al. (2012) reported that past and present human and natural disturbance and vegetation succession can mimic, exacerbate, counteract, or mask the effects of climate change on streamflow, even in reference basins. Liu et al. (2024) found that nutrient export rates in forest‐dominated small watersheds vary substantially based on local climate and landscape conditions and that coupling these factors with a forest growth model is necessary for realistic assessment of nutrient dynamics and forest best management practice (BMP) effectiveness. Amatya et al. (2016) stressed that reference sites do change over time in response to natural and anthropogenic disturbances (e.g., windthrow, insects, fire, hurricanes, climatic change, atmospheric pollution, and invasive species) but that these responses are typically minor compared to experimental treatments and disturbances designed to mimic anthropogenic and natural disturbances. Consequently, understanding temporal changes in nutrient concentrations in reference streams is vital for managing forest and water resources (Argerich et al., 2013).

The USDA Forest Service Experimental Forest and Range Network was progressively established beginning in 1908 (Neary et al., 2012). The network consists of more than 80 sites across the continental United States, Puerto Rico, and Alaska and has produced a key national repository of long‐term data from ∼20 least‐impaired (reference), managed, and disturbed watersheds (https://research.fs.usda.gov/forestsandranges). Data from the network were used by Argerich et al. (2013) in their comparison of proposed nutrient criteria to the range and variability of nutrients in reference watersheds. In addition, Ryan (2022) synthesized nutrient data and biological nutrient responses in streams draining multiple watersheds from Forest Service long‐term experimental forests and ranges in 15 different ecoregions to inform establishment and enforcement of water quality regulations. The objectives of most of the 19 chapters in Ryan (2022) were to (1) synthesize how the existing research and monitoring findings from individual experimental forests and ranges could be used by the water resource community for practical applications, and/or (2) identify gaps in the existing science at each experimental forest and range site that, if filled, would benefit regulatory and management agencies. Additional analyses of in‐steam nutrient data are needed to quantify the range and variability of background sediment and nutrients. It is anticipated that future analyses utilizing these data will increase in frequency and importance because of their relevance and widespread recognition as a reliable source of peer‐reviewed, scientifically sound data.

In contrast to large‐scale (river basin) watershed processes and water quality from headwater forest reference watersheds, sediment and nutrient runoff from grassland reference watersheds have received much less research attention. In fact, to our knowledge, no comprehensive analysis or compilation of background water quality has ever been published on native grasslands at the “micro‐watershed,” edge‐of‐field, or small watershed scale, which are the scales at which homogenous land uses, biological, and physical settings typically exist (Huntington et al., 1996); however, it is at that scale that land use export coefficients for background water quality are assigned to similar land uses in decision support tools and models and used for comparative analysis of pollutant sources (Amatya et al., 2004; Reckhow et al., 1980; Wickham et al., 2003). Thus, given the value of these reference water quality data in decision‐making, policy formulation, and land management, the objectives of the present work were to (1) summarize available sediment, N, P, and runoff from small grassland reference watersheds and forest reference watersheds, and (2) perform a cursory comparison between water quality from these two reference land uses. We also briefly discuss the value of reference water quality data in future analyses, decision‐making, policy formulation, and land management.

## 2 MATERIALS AND METHODS

To compile the most comprehensive listing of reference water quality data possible, we explored available databases and literature sources, including:

1. The “Measured Annual Nutrient loads from Agricultural Environments” (MANAGE) database (Harmel et al., 2006, 2022; Hopkins et al., 2025) expanded a key 1980s compilation of nutrient export data (Beaulac, 1980; Beaulac & Reckhow, 1982; Reckhow et al., 1980). MANAGE provides a readily accessible repository of site characteristic and field‐scale nutrient export data for agricultural lands (i.e., cultivated, grassland, and forest) (https://agdatacommons.nal.usda.gov/articles/dataset/Measured_Annual_Nutrient_loads_from_AGricultural_Environments_MANAGE_database/24660720).
2. The Forest Service Research Data Archive contains ecohydrology data and metadata, including nutrients and sediment for selected experimental forest and range watersheds (www.fs.usda.gov/rds/archive).
3. The USDA Forest Service Experimental Forest and Range Network StreamChemDB previously provided access to long‐term stream water chemistry data records and associated metadata, but now the following link provides a list of experimental forests that report water quality data with links to those data if available (www.smartforests.org/efr_water_quality).
4. The Environmental Data Initiative contains data from reference forest sites (https://edirepository.org).

Data were only included for homogeneous land use sites, either forested or grassland. It is important to note that “reference” sites can include a variety of biological conditions, including historical, least disturbed, minimally disturbed, and best attainable (Stoddard et al., 2006); therefore, the decision to include or exclude sites in the present analysis certainly involved some subjectivity. We used site descriptions in the publications and/or expert knowledge of scientists involved in studies at the sites to guide those decisions. For grassland reference sites, we included data from undisturbed remnant grasslands and minimally disturbed grasslands (e.g., low/moderate grazing intensity and managed rotational grazing) but did not include data from grassland sites with heavy grazing and/or fertilizer application. For forest reference sites, we included data only from the minimally disturbed or undisturbed forests at the USDA Forest Service long‐term experimental forests.

**FIGURE 1:**
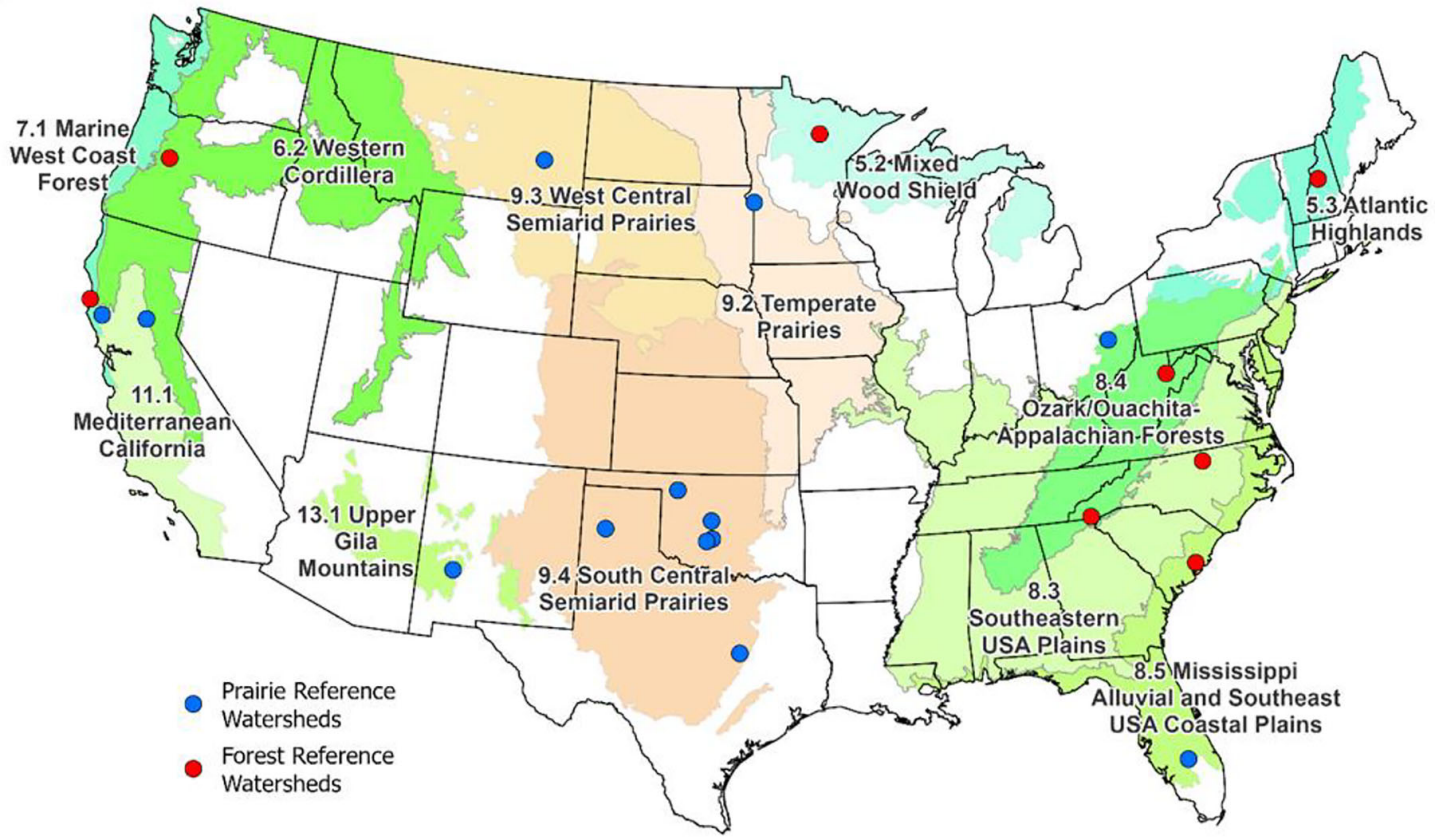
Reference grassland and forested watersheds with published runoff, sediment, N, and P runoff data, shown with the Level II US ecoregions (Omernik, 1987; Omernik & Griffith, 2014).

For each reference watershed (Figure 1), we compiled data on general characteristics, including location (state), watershed ID (name), drainage area, Level II ecoregion, and dominant soil series (Tables 1 and 2). We then calculated basic descriptive statistics for hydrologic characteristics (i.e., precipitation, potential evapotranspiration (PET), dryness index, runoff, and runoff coefficient) and for water quality constituent loads in runoff (sediment, dissolved N and P, particulate N and P, and total N and P) for each reference watershed (Tables 3 and 4).

**TABLE 1:** Summary information for grassland reference watershed sites with runoff water quality data.

Site name	Watershed name(s)	Lat/long coordinates	State	Level II ecoregion	Drainage area (ha)	Dominant soil series	Data available
USDA‐ARS North Appalachian Experimental Watershed (Owens et al., 1983)	Ungrazed pasture	40.37 N, −81.79 W	Ohio	8.4 Ozark‐Ouachita, App. Forests	28.2	Middle Kittanning clay	Runoff (1975–1976) Sediment (1975–1976) N (1975–1976) P (1975–1976)
University of Florida MacArthur Agroecology Research Center (Capece et al., 2007)	Winter pasture high	27.15 N, −81.20 W	Florida	8.5 Miss. Alluvial, Southeast USA Coastal Plains	32.4	Pineda fine sand	Runoff (1999–2003) P (1999–2003)
Big Stone County (Timmons & Holt, 1977)	Native prairie plots	45.42 N, −96.44 W	Minnesota	9.2 Temperate Prairies	0.009	Barnes silt loam	Runoff (1970–1974) N (1970–1974) P (1970‐1974)
USDA‐ARS Fort Keogh Livestock and Range Research Laboratory (Emmerich & Heitschmidt, 2002)	Ungrazed, 94 grazed, 94–95 grazed	46.38 N, −105.88 W	Montana	9.3 West‐Central Semi‐Arid Prairies	0.005	Kobase silty clay loam	Sediment (2005) N (2005) P (2005)
USDA‐ARS Riesel Watersheds (Harmel et al., 2004, 2009; S. J. Smith et al., 1983)	SW12	31.48 N, −96.89 W	Texas	9.4 South‐Central Semi‐Arid Prairies	1.2	Houston Black clay	Runoff (1938–2023) Sediment (2000–present) N (2000–present) P (2000–present)
USDA‐ARS Chickasha Watersheds (Menzel et al., 1978; Olness et al., 1980)	R5, R6	35.02 N, −97.99 W	Oklahoma	9.4 South‐Central Semi‐Arid Prairies	2.7	Renfrow, Grant, and Kingfisher silt loam	Runoff (1972–1976) Sediment (1972–1976) N (1972–1976) P (1972–1976)
USDA‐ARS Cyril Watershed (S. J. Smith et al., 1991)	5233	34.93 N, −98.20 W	Oklahoma	9.4 South‐Central Semi‐Arid Prairies	2.3	Lucien and Minco silt loam	Runoff (1980–1982) Sediment (1980–1982) N (1980–1982) P (1980–1982)
USDA‐ARS El Reno Watersheds (Sharpley et al., 1991; S. J. Smith et al., 1983)	E1–E4, FR3, FR5–FR8	35.56 N, −98.04 W	Oklahoma	9.4 South‐Central Semi‐Arid Prairies	1.6	Kirkland and Bethany silt loam	Runoff (1977–1992) Sediment (1977–1992) N (1977–1980) P (1977–1992)
USDA‐ARS Woodward Watersheds (Sharpley et al., 1991; S. J. Smith et al., 1983; Berg et al., 1988)	WW1–WW4	36.49 N, −99.37 W	Oklahoma	9.4 South‐Central Semi‐Arid Prairies	2.7–5.6	Woodward and Quinlan loam	Runoff (1977–1992) Sediment (1977–1992) N (1977–1992) P (1977–1992)
USDA‐ARS Bushland Watersheds (Jones et al., 1985; Sharpley et al., 1991; S. J. Smith et al., 1983, 1991)	N/S, NG, SG	35.19 N, −102.08 W	Texas	9.4 South‐Central Semi‐Arid Prairies	0.04–3.3	Pullman clay loam	Runoff (1978–1983) Sediment (1978–1983) N (1978—1983) P (1978‐1983
Univ. California Sierra Foothill Research and Extension Center (D. L. Lewis et al., 2006; Tate et al., 1999)	Schubert watershed	39.25 N, −121.31 W	California	11.1 Mediterranean California	103	Auburn and Sobrante silt loam	Sediment (1981–1988) N (1980–2000) P (1981–1988)
University of California Hopland Research and Extension Center (Dahlgren et al., 2001)	C	39.00 N, −123.08 W	California	11.1 Mediterranean California	22.7	Yorkville and Hopland loam	Runoff (1999) Sediment (1999) N (1999) P (1999)
Sierra County (Cabrera et al., 2009)	Control	33.47 N, −107.70 W	New Mexico	13.1 Upper Gila Mountains	0.01	Ildefonso sandy loam	Runoff (2000—2002) Sediment (2000‐2002) P (2000–2002)

**TABLE 2:** Summary information for forest reference watershed sites with runoff water quality data.

Site name	Watershed name(s)	Lat/long coordinates	State	Level II ecoregion	Drainage area (ha)	Dominant soil series	Data available
Marcell Experimental Forest (Verry et al., 2018; Sebestyen et al., 2021, 2022)	S2, S5	47.53 N, −93.46 W	Minnesota	5.2 Mixed Wood Shield	9.7, 52.6	Warba very fine sandy loam, Nashwauk fine sandy loam	Streamflow (1961–2016) N (1992–2016) P (1986–2016)
Hubbard‐Brook Experimental Forest (Campbell et al., 2021; Likens, 2010)	W6	43.94 N, −71.75 W	New Hampshire	5.3 Atlantic Highlands	13.2	Tunbidge‐Lyman, Berkshire, and Skerry sandy loam	Sediment (1965–present) N (1964–present) P (1972–present)
H. J. Andrews Experimental Forest (Daly et al., 2019; S. L. Johnson & Fredriksen, 2019; S. L. Johnson et al., 2021; S. Johnson et al., 2023)	WS02	44.23 N, −122.17 W	Oregon	6.2 Western Cordillera	60	Aschoff‐Cascadia Complex loam and clay loam, Lempira gravelly loam	Runoff (1952–present) Sediment (1981–present) N (1981–present) P (1981–present)
Caspar Creek Experimental Watershed (Dymond et al., 2021)	MUN, WIL	39.36 N, −123.73 W	California	7.1 Marine West Coast Forest	15, 26	Irmulco‐Tramway complex, Vandamme Loam	Streamflow (1986–2024) Sediment (1986–2024) N (1991–1996, 2016–2020) P (1991–1996, 2016–2020)
Hill Forest Experimental Forest (Boggs et al., 2015, 2020)	HF2, HFW2	36.22 N, −78.87 W	North Carolina	8.3 Southeastern USA Plains	12, 40	Fine, mixed, semiactive, thermic Typic Hapludults, loam to sandy loam	Runoff (2008–present) Sediment (2008–2013) N (2008–2013) P (2008–2013)
Umstead Research Farm (Boggs et al., 2015, 2020)	UF2	36.18 N, −78.78 W	North Carolina	8.3 Southeastern USA Plains	29	Fine, mixed, semiactive, thermic Aquic Hapludult, loam to sandy loam	Runoff (2008–present) Sediment (2008–2013) N (2008–2013) P (2008–2013)
Coweeta Hydrology Lab Experimental Forest (Swank et al., 2014; Caldwell et al., 2016; Brown et al., 2021a)	WS2	35.05 N, −83.45 W	North Carolina	8.4 Ozark‐Ouachita, App. Forests	12.3	Mesic Typic Dystrochrepts or Hapludults (hillslopes), Mesic Typic Haplumbrepts (riparian)	Runoff (1937–present) Sediment (1974–1976) N (1972–present) P (1972–present
Fernow Experimental Forest (Adams et al., 1994, 2012)	WS4	39.06 N, −79.67 W	West Virgina	8.4 Ozark‐Ouachita, App. Forests	39	Calvin, Ernest, and Dekalb channery loam	Runoff (1951–present) Sediment (1981–2010) N (1981–present)
Santee Experimental Forest (Amatya et al., 2006, 2022; Muwamba et al., 2016; Trettin et al., 2022; Walega et al., 2024)	WS80	33.15 N, −79.8 W	South Carolina	8.5 Miss. Alluvial, Southeast USA Coastal Plains	160	Wahee clay loam	N (1976–1981; 1990–1994; 2003–2023) P (1976–1981; 1990–1994; 2003–2023)

**TABLE 3:** Average annual hydrologic characteristics and sediment, N, and P loads for each grassland reference watershed site, presented in the order of ascending mean annual rainfall.

Reference watershed site	Rainfall^a^ (mm/year)	PET^b^ (mm/year)	Dryness index (PET/rainfall)	Runoff^a^ (mm/year)	Runoff coefficient	Sediment^a^ (kg/ha/year)	Diss. N^a^ (kg/ha/year)	Part. N^a^ (kg/ha/year)	Total N^a^ (kg/ha/year)	Diss P^a^ (kg/ha/year)	Part. P^a^ (kg/ha/year)	Total P^a^ (kg/ha/year)
Sierra County, New Mexico (12 years)	242	661	2.73	<1	0.00	8	–	–	–	–	–	<0.01
USDA‐ARS Fort Keogh Livestock and Range Research Laboratory (6 years)	284	674	2.37	–	–	3	–	0.01	–	–	<0.01	–
USDA‐ARS Bushland Watersheds (3–18 years)	446	868	1.95	10	0.01	55	0.15	0.43	0.42	0.08	0.03	0.10
Big Stone County, Minnesota (15 years)	595	631	1.06	30	0.05	150^c^	0.12	0.57	0.84	0.02	0.09	0.11
USDA‐ARS Woodward Watersheds (30–62 years)	650	918	1.41	12	0.02	349	0.03	–	0.51	0.03	–	0.13
University of California Sierra Foothill Research and Extension Center (18–20 years)	734	911	1.24	353	0.48	197	1.60	–	1.60	0.00^d^	–	0.03
USDA‐ARS El Reno Watersheds (16–76 years)	740	949	1.28	90	0.12	37	0.60	–	0.80	0.04	–	0.21
USDA‐ARS Cyril Watershed (3 years)	790	981	1.26	5	0.01	43	0.02	–	0.11	0.01	–	0.02
University of California Hopland Research and Extension Center (1 year)	912	835	0.92	117	0.13	76	0.10	–	–	0.01	–	–
USDA‐ARS Chickasha Watersheds (7–14 years)	934	986	1.06	89	0.10	542	0.44	1.47	1.90	0.03	0.50	0.70
USDA‐ARS North Appalachian Experimental Watershed (2 years)	991	711	0.72	123	0.12	228	0.80	0.60	0.14	–	–	0.10
USDA‐ARS Riesel Watersheds (12 years)	994	1119	1.13	157	0.16	507	1.63	0.61	2.23	0.12	0.10	0.36
Univ. Florida MacArthur Agroecology Research Center (10 years)	1225	1258	1.03	237	0.19	–	–	–	–	–	–	0.37

^a^ Annual means for precipitation, runoff, and sediment and nutrient loads as reported by studies in the MANAGE database (Harmel et al., 2022).

^b^ Potential ET (PET) was calculated with the widely used, simple, and reliable Hamon method that uses air temperature and potential daytime length (Sun et al., 2011). See DeJonge et al. (2025) for a definition and understanding of PET as applied.

^c^ The only sediment runoff data obtained for the Temperate Prairies were from a 1 year study by Miller et al. (2021).

^d^ Based on 1 site‐year of data.

**TABLE 4:** Average annual hydrologic characteristics and sediment, N, and P loads for each forest reference watershed site, presented in the order of ascending mean annual rainfall.

Reference watershed site	Rainfall (mm/year)	PET^a^ (mm/year)	Dryness index (PET/rainfall)	Runoff (mm/year)	Runoff coefficient	Sediment (kg/ha/year)	Diss. N (kg/ha/year)	Part. N (kg/ha/year)	Total N (kg/ha/year)	Diss P (kg/ha/year)	Part. P (kg/ha/year)	Total P (kg/ha/year)
Marcell Experimental Forest (S2; 20 years total N, 32 years total P)	875	534	0.61	161	0.18	–	1.02	–	–	–	–	0.09
Marcell Experimental Forest (S5; 20 years total N, 27 years total P)	875	534	0.61	108	0.12	–	0.64	–	–	–	–	0.04
Hill Forest Experimental Forest (HF2; 6 years)	1180	877	0.74	208	0.18	70	0.13	–	1.60	–	–	0.18
Hill Forest Experimental Forest (HFW2; 6 years)	1180	877	0.74	180	0.15	50	0.11	–	1.20	–	–	0.19
Umstead Research Farm (6 years)	1180	877	0.74	245	0.21	80	0.29	–	2.30	–	–	0.19
Caspar Creek Experimental Watershed (MUN; 6 years)^b^	1111	639	0.53	363	0.33	256	–	–	0.03	–	–	<0.01
Caspar Creek Experimental Watershed (WIL; 20 years)^c^	1111	639	0.53	519	0.47	169	1.00	–	1.22	–	–	0.30
Hubbard‐Brook Experimental Forest (61 years)	1228	551	0.45	939	0.76	40	1.70	–	–	0.01	–	–
Santee Experimental Forest (20 years)^d^	1403	1036	0.74	253	0.18	–	1.45	–	–	0.06	0.05	0.10
Fernow Experimental Forest (30–72 years)^e^	1450	633	0.44	695	0.48	20	4.63	–	–	–	–	–
Coweeta Hydrology Lab Experimental Forest (2 years sediment, 46 years N, P)	1849	879	0.49	819	0.44	140	0.07	–	–	0.05	–	–
H.J. Andrews Experimental Forest (38 years)	2100	576	0.27	1274	0.61	380	0.36	0.47	0.84	0.40	0.17	0.54

^a^ Potential ET (PET) was calculated with widely used, simple, and reliable Hamon method that uses air temperature and potential daytime length (Sun et al., 2011), except for Coweeta site where pan evaporation was used. See DeJonge et al. (2025) for a definition and understanding of PET as applied.

^b^ Caspar Creek (MUN) sediment data is suspended load (J. Lewis et al., 2001); nutrient data as reported by Dahlgren (1998) for water years 1991–1996.

^c^ Caspar Creek (WIL) sediment data is suspended load (unpublished); nutrient data compiled from Dahlke (2021) for water years 2016–2020.

^d^ Santee nutrient data (Trettin et al., 2022; Walega et al., 2024) reported only for 2003–2024. Earlier periods with extensive data gaps (1976−1981 and 1990–1994) were excluded. The hydrologic impact of Hurricane Hugo in September 1989 damaged >80% forest canopy and impacted baseline levels through 2004 (Jayakaran et al., 2014).

^e^ For the Fernow Experimental Forest, rainfall data are available from 1951−2022, runoff data from 1951–2021, sediment data from 1981−2010, dissolved N data from 1971−2021.

### 2.1 Estimation of annual loads

For grassland reference sites, mean annual sediment and nutrient loads were acquired from the relevant publications (Table 3). In contrast, mean annual sediment and nutrient loads for the forest reference sites (Table 4) were calculated as described subsequently. For the Santee Experimental Watersheds, the “backward” method was used to calculate the load (i.e., same daily averaged flow proportional concentration, assumed for each day of the period from last sample to the current sample, was multiplied with the daily flow, integrated using flow rates calculated from 15‐min stage measurements, and summed for the period). Then the load for each period was summed for the year. For the Caspar Creek Experimental Watersheds, annual nutrient loads were calculated by multiplying the average concentrations of the previous and current samples by the cumulative streamflow between samples, with more frequent sampling during storm events. For Caspar Creek, sediment loads were estimated by the Turbidity Threshold Sampling method (J. Lewis & Eads, 2009) where continuous in‐stream turbidity data are used to control automated sampling, and event‐specific turbidity‐sediment rating curves are then developed to estimate unsampled concentrations that are then summed for total load. For the Fernow and Coweeta watersheds, annual loads were calculated by summing the cumulative streamflow multiplied by nutrient concentrations in a weekly stream grab sample (Brown et al., 2021b). At the Hill Forest and Umstead, annual loads were obtained by summing monthly cumulative streamflow multiplied by monthly nutrient concentrations in stream grab samples. At H. J. Andrews, flow‐proportional water samples were collected over a 3‐week period, and loads were calculated using average proportional concentrations multiplied by the discharge for each period, then summed to obtain annual loads. At the Marcell Experimental Forest, daily streamflow and concentration values (extended backward and forward to the midpoint between adjacent samples) were multiplied and summed to obtain annual loads.

It is important to note that results from meta‐type analyses are impacted by differences in field and analytical methods (Eagle et al., 2017). Specifically for the present study, reported sediment and nutrient loads were no doubt influenced by differing sample collection methods and frequencies and by differing analytical procedures, protocols, detection limits, analytes, and load estimation techniques, none of which were explicitly considered in the calculations.

## 3 RESULTS AND DISCUSSION

### 3.1 Grassland reference sites

Measured annual nutrient runoff load data from 13 grassland reference sites comprised of 23 watersheds (or treatment watershed groups) were compiled. These sites were located in seven of the 50 North American Level II ecoregions (Table 1; Figure 1); however, extensive data were only available in the South‐Central Semi‐Arid Prairies (11 studies with 68–178 site‐years) and in Mediterranean California (three studies with 18–20 site‐years) (Table 1). The remaining five ecoregions with data (Ozark‐Ouachita, Appalachian Forests; Mississippi Alluvial, Southeast USA Coastal Plains; Temperate Prairies; West‐Central Semi‐Arid Prairies; and Upper Gila Mountains) have only one study with less than 20 site‐years each. As expected for homogeneous (single land use) sites, watershed sizes for the grassland reference sites were quite small with several micro‐watersheds (<0.1 ha), several in the 1–10 ha range, and only four larger than 20 ha (Table 1).

As expected, the grassland watersheds were relatively arid (average precipitation = 733 mm/year), and most had dryness indices (PET/precipitation) >1.0 indicating water limitations (Table 3). Correspondingly, runoff was often relatively low with five watersheds averaging 0–12 mm of annual runoff, although five had runoff that exceeded 100 mm/year. Annual runoff coefficients (runoff/precipitation) ranged from <0.005 to 0.19 due to differing slopes, slope lengths, soils, vegetation, and climatic conditions. An interesting anomaly in runoff occurred at the University of California Sierra Foothill Research and Extension Center, with only 734 mm of annual rainfall but the highest average runoff (353 mm/year) and runoff coefficient (0.48) of any grassland reference watershed. The California Annual Grasslands are a unique land type as they are dominated by non‐native annual grasses that originated from Spanish colonization (1700s) supported by winter precipitation patterns associated with the Mediterranean climate (Corbin & D'Antonio, 2004; Seabloom et al., 2003). The divergence between precipitation timing and plant growth as well as the prevalence of annual grasses in this region likely contribute to greater than expected runoff. Alternatively, the greater runoff in some regions may be due to the influence of return flows in larger headwater streams (Gebert et al., 1987). For example, R. A. Smith et al. (2003) reported ranges in the 90th percentile of annual average runoff of 50–1100 mm/year in the Western Forested Mountains, whereas annual runoff ranged from <1 to 353 mm/year in roughly the same region (Mediterranean California and Upper Gila Mountain ecoregions) in the present analysis (Table 3). However, R. A. Smith et al. (2003) reported similar runoff ranges for the Great Plains/Shrublands (20–30 mm/year) and the cultivated Great Plains (20–110 mm/year) as we found in the present analysis for the South‐Central Semi‐Arid Prairies ecoregion (10–157 mm/year).

Annual sediment loads were also quite low with six watersheds averaging <100 kg/ha and only two watersheds averaging >500 kg/ha (Table 3). While the focus of the present study is reference runoff water quality, several of the reference watersheds that were “minimally impacted” with light grazing also had companion watersheds that were impacted by heavy grazing. Although data from these impacted sites were not included in the present analysis, these companion watersheds clearly highlighted the importance of proper grazing management to limit soil erosion. A dramatic example of overgrazing impacts occurred at the USDA‐ARS Chickasha Watersheds where Watersheds R5 and R6 were rotationally grazed at a moderate intensity and Watershed R8 (not included as a reference watershed) was grazed continuously with excessive stocking rates (Table 3). The excessive stocking rate produced much higher annual sediment loads (>8000 kg/ha) and almost twice the runoff (170 mm) as the moderate stocking rate on the reference grassland watersheds (Menzel et al., 1978). Watershed R8 also had extensive active gullies on ∼5% of the watershed area; thus, the cause and effect related to overgrazing is not clear.

Mean annual N loads from small reference grassland watersheds exhibited substantial variability. Dissolved and total N loads were lowest at the USDA‐ARS Cyril Watersheds (0.02 and 0.11 kg/ha/year) and were highest at the USDA‐ARS Riesel Watersheds (1.63 and 2.23 kg/ha/year). It is interesting that these loads from reference micro‐watersheds are similar in magnitude and range as from reference streams and rivers draining larger watersheds based on R. A. Smith et al. (2003), who reported ranges in median annual total N loads from 0.01 kg/ha/year in the xeric west to 2.2 kg/ha/year in the region similar to the Mississippi Alluvial, Southeast USA Coastal Plains ecoregion. The highest average annual particulate N load (1.47 kg/ha) occurred at the USDA‐ARS Chickasha Watersheds, which is not surprising since that site also had the largest annual average sediment load (542 kg/ha).

Mean annual dissolved and particulate P loads from small reference grassland watersheds were lower and showed less variability. Dissolved P loads ranged from 0.00 to 0.12 kg/ha/year, and none of the particulate P loads exceeded 0.10 kg/ha/year except for the USDA‐ARS Chickasha Watersheds (0.50 kg/ha/year). Total P loads were more variable, ranging from <0.01 kg/ha/year in Sierra County, New Mexico to 0.70 kg/ha/year at the USDA‐ARS Chickasha Watersheds. At several reference grassland watersheds (e.g., USDA‐ARS Riesel Watersheds, 0.36 kg/ha/year; University of Florida MacArthur Agroecology Research Center, 0.37 kg/ha/year; USDA‐ARS Chickasha Watersheds, 0.70 kg/ha/year), total P loads exceeded the ranges reported by R. A. Smith et al. (2003), who reported ranges in background median total P loads in streams and rivers from <0.01 kg/ha/year in the xeric west to 0.06 kg/ha/year in the cultivated Great Plains.

While the focus of the present analysis is on sediment, N, and P loads, constituent concentrations are also valuable in terms of reference water quality. For the South‐Central Semi‐Arid Prairies ecoregion, which contains the most reference grassland watersheds, Nelson et al. (2020) presented a summary of concentration data from many of the same edge‐of‐field studies that are included in the present analysis. Nelson et al. (2020) reported total Kjeldahl N concentrations from 0.1 to 575 mg/L (median = 1.8 mg/L) and total P concentrations from >0.0001 to 4.0 mg/L (median = 0.192 mg/L). R. A. Smith et al. (2003) reported total N concentrations from 0.04 to 0.50 mg/L (median = 0.30 mg/L) and total P concentrations from 0.012 to 0.08 mg/L (median = 0.055 mg/L) from the cultivated Great Plains, which is similar to the South‐Central Semi‐Arid Prairies ecoregion in terms of rainfall and land management. These results support the expectation that nutrient concentrations decrease as watershed size increases; however, the reduction is due to in‐stream processes that reduce nutrient mass (R. A. Smith et al., 2003; Birgand et al., 2007), not simply due to dilution because both represent reference sites.

### 3.2 Forest reference sites

Across forest reference sites, measured annual nutrient runoff loads were available from 12 reference watersheds at nine experimental forests (Table 2; Figure 1), with Marcell, Hill Forest, and Caspar Creek, each providing data from two reference watersheds. The reference watersheds with runoff and water quality data are located in seven of the 50 North American Level II ecoregions (Mixed Wood Shield; Atlantic Highlands; Western Cordillera; Marine West Coast Forest; Southeastern USA Plains; Ozark‐Ouachita, Appalachian Forests; Mississippi Alluvial, Southeast USA Coastal Plains). These reference watersheds ranged in size from 9.7 ha for S2 in the Marcell Experimental Forest to 160 ha for WS80 in Santee Experimental Forest, but most were in the 10–50 ha range.

Most forested sites were in humid regions with annual precipitation ranging from 875 to 2100 mm and runoff ranging from 108 to 1274 mm (Table 4). Annual runoff coefficients ranged from 0.12 to 0.76 due to differing slopes, slope lengths, soils, vegetation, and climatic conditions. The H. J. Andrews Experimental Forest in the seasonably wet western portion of the Western Cordillera ecoregion had the highest precipitation (2100 mm/year) and runoff (1274 mm/year) and the lowest dryness index (0.27), while the Marcell Experimental Forest in the Mixed Wood Shield ecoregion had the lowest precipitation (875 mm/year) and runoff (108 mm/year). The Santee Experimental Forest in the humid Mississippi Alluvial, Southeast USA Coastal Plains and the Hill Forest and Umstead sites in the Southeastern USA Plains had dryness indices of 0.74 due to much higher PET values. The variability in annual runoff was relatively high with five watersheds averaging >500 mm of annual runoff but with three watersheds averaging <200 mm, which is attributed to substantial differences in climate and topography, although vegetation and soil may also be the factors (Sun et al., 2002). The low‐gradient Santee Experimental Forest and the Marcell Experimental Forest watersheds had relatively low runoff relative to precipitation, while the high‐gradient Hubbard‐Brook Experimental Forest yielded the highest runoff (76% of mean precipitation), followed by the H. J. Andrews Experimental Forest (61%). See Amatya et al. (2016) for additional information on the seasonal daily runoff and daily flow duration curves for many of these watersheds, and see Amatya et al. (2025) for trends and characteristics of hydroclimatic extremes for most of these watersheds.

Sediment runoff load data were available for nine of the 12 reference watersheds, with the low gradient Marcell and Santee sites not reporting sediment data. Mean annual sediment loads ranged from 20 kg/ha at the Fernow Experimental Forest to 380 kg/ha at the H. J. Andrews Experimental Forest due to high topographic gradient and high runoff (Table 4). This is consistent with Schoenholtz (2004) and Hawkins et al. (2019), who noted that even in forested watersheds not subject to direct human disturbances, erosion rates are often highly spatially and temporally variable due to effects of natural events such as large storms, landslides, and fires and possibly previous human disturbances.

All of the reference forested watersheds reported dissolved N loads, with several low annual values (<0.15 kg/ha, Table 4). There were also several watersheds with relatively high annual dissolved N loads (e.g., 4.63 kg/ha at the Fernow Experimental Forest, 1.70 kg/ha at the Hubbard‐Brook Experimental Forest, and 1.45 kg/ha at the Santee Experimental Forest). The relatively high dissolved N loads at the Fernow, Hubbard‐Brook, and Santee watersheds are consistent with Binkley et al. (2004), who reported that nitrate‐N dominated total N loads of streams draining hardwood forests, whereas dissolved organic N dominated the streams in coniferous forests. It is interesting that Caspar Creek, which is dominated by redwood and Douglas fir, had moderately large dissolved N loads, since Reid and Cafferata (2021) noted that stream water in Caspar Creek reference watersheds usually yielded nitrate concentrations less than the detection limit (0.02 mg/L) with maximum observed concentrations of 0.7 mg/L during storms. Other forms of dissolved N (e.g., nitrite, ammonia, ammonium, and organic N) might have also influenced these higher loads. Schoenholtz (2004) noted that conifer forests (such as the Santee Experimental Forest) tend to have more dissolved N in the organic form and hardwood forests (such as Fernow and Hubbard‐Brook Experimental Forests) tend to have more dissolved N in the inorganic form. The high variability of dissolved N loads among these forested watersheds may also be influenced by factors such as vegetation type and age, leaf fall, geologic substrate, stream order, basin size and morphology, atmospheric deposition, and climate (Schoenholtz, 2004). In fact, the unusually high stream N levels at the Fernow Experimental Forest are attributed to atmospheric deposition because of its location downwind of industrial centers in the US Midwest. In contrast to dissolved N loads, only one site reported particulate N loads and only six reported total N loads. Annual total N loads were less variable than dissolved N with most values between 0.84 and 2.30 kg/ha. Interestingly, however, Caspar Creek watersheds produced the lowest total N load (MUN, 0.03 kg/ha) and a much higher load (WIL, 1.22 kg/ha) (Table 4), but this may be attributed to a more recent timber harvest in WIL (1970).

Dissolved P and total P loads from the reference forested watersheds were less variable (Table 4). The H. J. Andrews Experimental Forest yielded the highest annual dissolved P loads (0.40 kg/ha), and the rest were ≤0.06 kg/ha. H. J. Andrews (0.54 kg/ha) and watershed WIL at Caspar Creek (0.30 kg/ha) yielded the highest annual total P loads with other watersheds producing loads <0.20 kg/ha. As noted for total N loads, the impact of disturbance (timber harvest) is likely seen in the variability of total P loads in Caspar Creek watersheds (MUN, <0.01 kg/ha; WIL, 0.30 kg/ha). Only the H. J. Andrews Experimental Forest and Santee Experimental Forest reported particulate P loads.

### 3.3 Comparing grassland and forest reference sites

In general, the grassland reference watersheds received less rainfall (242–1225 mm/year) and had less runoff (<1–237 mm/year) than the forested reference watersheds with rainfall ranging from 875 to 2100 mm/year and runoff from 108 to 1274 mm. Several grassland and forested reference watersheds had very low annual sediment loads (<100 kg/ha), but sediment loads from the grassland reference watersheds (3–542 kg/ha/year) tended to be higher than from the forested reference watersheds (40–380 kg/ha/year). This was true in the Ozark‐Ouachita Appalachian Forests ecoregion with sediment load data from both a reference grassland and forest watershed. Although the grassland reference watersheds at the USDA‐ARS North Appalachian Experimental Watersheds had lower runoff (123 mm/year) than the Fernow Experimental Forest and Coweeta Hydrology Lab Experimental Forest (695–819 mm/year), they had higher annual sediment loads (228 kg/ha vs. 20–140 kg/ha). At the Fernow Experimental Forest this is attributed to extremely stony soil and high infiltration rates, which limit surface runoff. At the Marcell Experimental Forest, which is dominated by peatlands (wetlands), it is assumed that sediment load is so low that measurements are not taken. Schoenholtz (2004) reported that streams in forest lands produce very low sediment yield compared to other rural land uses (e.g., cropland) and that stream channels are likely the dominant contributor of suspended sediment in undisturbed forested watersheds (unless in geologically unstable terrain prone to landsliding). In addition, most forest soils have infiltration capacities that exceed common rainfall intensities; therefore, surface runoff and erosion are often relatively insignificant in undisturbed forested catchments (Schoenholtz, 2004).

The annual dissolved N loads from the grassland reference watersheds (0.02–1.63 kg/ha) tended to be lower than from the forest reference watersheds (0.07–4.63 kg/ha), despite rapid nutrient uptake by forest ecosystem biota (Schoenholtz, 2004). In the Ozark‐Ouachita Appalachian Forests ecoregion, the dissolved N load (0.80 kg/ha) at grassland reference watershed at the USDA‐ARS North Appalachian Experimental Watersheds was within the range of dissolved N loads (0.07–4.63 kg/ha) from the reference forested watersheds at the Fernow Experimental Forest and Coweeta Hydrology Lab Experimental Forest. In contrast, the total N loads for the grassland reference watersheds (0.11–2.23 kg/ha) were similar to those from the forested reference watersheds (0.03–2.30 kg/ha).

Annual total P loads from the grassland reference watersheds (<0.01–0.70 kg/ha) were also similar to those of the forested reference watersheds (<0.01–0.54 kg/ha). In contrast, in the Mississippi Alluvial Southeast Coastal Plains, total P loads (0.37 kg/ha/year) from the grassland reference watershed at the University of Florida MacArthur Agroecology Research Center were higher than from the Santee Experimental Forest (0.10 kg/ha), even though the sites have similar rainfall and runoff.

### 3.4 Example applications of reference water quality data

Water resource decision support tools, policy formulation, and regulatory actions all make assumptions about the relative contribution of anthropogenic activities; therefore, reference water quality data are necessary to better understand the effects of land use and management and to motivate mitigation of adverse impacts. Several examples appear subsequently.

1. Harmel et al. (2018) compared N and P runoff concentrations from a remanent native prairie reference site, a well‐managed grazed pasture, and a pasture with excessive nutrient application. They compared mean nitrate‐N, ammonium‐N, and dissolved P concentrations in runoff and concluded that mean values did not appropriately differentiate reference, well‐managed, and poorly managed pastures and suggested that the 90th percentile of concentrations from the reference pasture would be a better standard of comparison.
2. The South Carolina Department of Health and Environmental Control (now the Department of Environmental Services) studied stream flow and nutrient concentration and load data from reference and managed (treatment) watersheds in the Santee Experimental Forest. They used these data along with atmospheric deposition data to establish numeric nutrient criteria for estuaries, rivers, and streams considering the “undisturbed baseline” as well as the impacts of urbanization in the landscape (Trettin et al., 2022).
3. Santee Experimental Forest reference site data were also used in a comprehensive analysis of flow and water quality in Charleston Harbor (TetraTech, 2008). This dataset provided a valuable context for natural background loadings from the headwaters to the estuary and was used in revisions to total maximum daily loads (TMDLs) completed for the Charleston Harbor (Lu et al., 2005) and the Cooper and Ashley Rivers (Cantrell, 2013), as required by the federal Clean Water Act section 303(d).
4. Some states apply a reference watershed approach using “unimpaired” watersheds to establish acceptable loading rates that are then applied to impaired watersheds to calculate TMDLs (American Farmland Trust, 2013).
5. Data from Hill Forest and Umstead Research Farm were used in the 2022 revision of the North Carolina Forest Service BMP manual and to support regional watershed modeling efforts conducted through the Upper Neuse River Basin Association's modeling initiative (Upper Neuse River Basin Association, 2016). Reference data from these forested watersheds allowed the users to isolate baseline hydrologic and water quality trends, which were then used to inform BMPs to manage water resources for both rural and urban forest systems. In addition, these data helped refine nutrient and carbon loading estimates for Falls Lake, a major drinking water reservoir, and provided guidance for the role of evapotranspiration in protecting water quality in a rapidly urbanizing region.

## 4 CONCLUSIONS

Reference watersheds can be pristine or minimally impacted, and the natural background contribution from reference grassland and forested watersheds is important to guide research, modeling, management, and policy. To our knowledge, this study is the first to compile and analyze reference water quality from native grasslands and to provide a cursory comparison of water quality from grassland and forest reference watersheds. In response to the need for these data, we compiled measured annual nutrient load data. Based on our extensive review, data were available from 13 grassland reference sites with 23 watersheds (or watershed groups) and from 12 forest reference watersheds at nine experimental forests across 12 North American Level II ecoregions (Tables 1, 2, 3, 4).

The grassland reference sites were relatively arid, whereas the forest reference sites were humid. Several grassland and forested reference watersheds had very low mean annual sediment loads, but a higher proportion of grassland reference watersheds had relatively high sediments loads. Annual dissolved N and P loads from the grassland reference watersheds tended to be lower than from forest reference watersheds despite rapid nutrient uptake by forest ecosystem biota. However, average total N and P loads were similar for grassland and forested reference watersheds.

One surprising observation was that most of the edge‐of‐field studies on reference grassland watersheds (12 of 13) and forested reference sites (9 of 12) measured sediment loss in runoff, which indicates that the authors viewed this as an important process. In contrast, studies such as R. A. Smith et al. (2003) did not address “background” sediment losses, likely due to high variability in natural geomorphological process and difficulty in differentiating natural and anthropogenic contribution in larger streams and rivers.

### 4.1 Future research

While the present research provides valuable summary results and initial comparisons related to reference water quality across the United States, additional research and in‐depth analyses are needed to guide policy formulation and programmatic decision‐making. Several research needs based on the current findings are presented subsequently.

1. W. M. Lewis et al. (1999) and Dodds and Welch (2000) noted that water chemistry, temperature, sediment, and flows can vary markedly between ecoregions and between reference watersheds in the same ecoregion. In the present study, watersheds within the Caspar Creek Experimental Watershed showed substantial differences, whereas watersheds at Hill Forest Experimental Forest had similar loads (Table 4); hence, future research is needed to better understand these differences in behavior. A “one‐size‐fits‐all” approach to establishing numeric criteria almost certainly would produce water‐quality or ecological standards that either are not realistically attainable given natural conditions (e.g., R. A. Smith et al., 2003) or are not protective enough (e.g., Dodds & Oakes, 2004).
2. Results of the present analysis provide important reminders that surface runoff from natural areas (reference sites) transports sediment, N, and P offsite, and sometimes in appreciable amounts. Research has shown that mismanaged anthropogenic activities on the land can dramatically increase these losses, but additional knowledge is needed on how best to account for natural (background) contributions in watershed plans, decision support tools, nutrient budgets, and hydraulic structure design.
3. Greathouse et al. (2012) reported challenges they encountered in a synthesis study of nutrient losses from eleven Forest Service Experimental Forests and Ranges, highlighting differences in (1) sampling and analytical methods, (2) forms of nutrients reported, (3) terminology and units, (4) sample collection timing, (5) detection limits, and (6) poor documentation (metadata) for historical data. Similarly, Eagle et al. (2017) noted that data (metadata) and reporting deficiencies reduce the effectiveness of agri‐environmental meta‐analysis. For these reasons and others, Harmel et al. (2021) stressed the importance of moving beyond data management to a comprehensive data stewardship ethic that addresses data collection, quality assurance‐quality control, processing, analysis, storage, transfer, visualization, and maintenance. Incorporating data stewardship into watershed and ecological research, including projects on reference watersheds, is an important research advancement.

## AUTHOR CONTRIBUTIONS

**R. Daren Harmel**: Conceptualization; data curation; formal analysis; investigation; writing—original draft; writing—review and editing. **Devendra Amatya**: Conceptualization; data curation; formal analysis; writing—original draft; writing—review and editing. **Stephen Sebestyen**: Data curation; writing—original draft; writing—review and editing. **Ge Sun**: Data curation; formal analysis; writing—original draft; writing—review and editing. **Joshua Mott**: Formal analysis; writing—original draft; writing—review and editing. **Merilynn Schantz**: Formal analysis; writing—original draft; writing—review and editing. **Johnny Boggs**: Data curation; writing—original draft; writing—review and editing. **Peter Caldwell**: Data curation; writing—original draft; writing—review and editing. **John Campbell**: Data curation; writing—original draft; writing—review and editing. **Aaron Hird**: Writing—original draft; writing—review and editing. **Elizabeth T. Keppeler**: Data curation; writing—original draft; writing—review and editing. **Ben Rau**: Data curation; writing—original draft; writing—review and editing. **Sherri L. Johnson**: Data curation; formal analysis; writing—original draft; writing—review and editing. **Doug Smith**: Data curation; writing—original draft; writing—review and editing.

## CONFLICTS OF INTEREST STATEMENT

The authors declare no conflicts of interest.
